# Distance Measurements in UWB-Radio Localization Systems Corrected with a Feedforward Neural Network Model

**DOI:** 10.3390/s21072294

**Published:** 2021-03-25

**Authors:** Peter Krapež, Matjaž Vidmar, Marko Munih

**Affiliations:** 1 Laboratory of Robotics, Faculty of Electrical Engineering, University of Ljubljana, Tržaška cesta 25, 1000 Ljubljana, Slovenia; marko.munih@fe.uni-lj.si; 2 Radiation and Optics Laboratory, Faculty of Electrical Engineering, University of Ljubljana, Tržaška cesta 25, 1000 Ljubljana, Slovenia; matjaz.vidmar@fe.uni-lj.si

**Keywords:** ultra-wideband technology, real-time localization system, distance measurement, error mitigation, tag orientation

## Abstract

An ultra-wideband (UWB) localization system is an alternative in a GPS-denied environment. However, a distance measurement with UWB modules using a two-way communication protocol induces an orientation-dependent error. Previous research studied this error by looking at parameters such as the received power and the channel response signal. In this paper, the neural network (NN) method for correcting the orientation-induced distance error without the need to calculate the signal strength, obtain the channel response or know any parameters of the antenna and the UWB modules is presented. The NN method utilizes only the measured distance and the tag orientation, and implements an NN model obtained by machine learning, using measurements at different distances and orientations of the two UWB modules. The verification of the experimental setup with 12 anchors and a tag shows that with the proposed NN method, 5 cm better root mean square error values (RMSEs) are obtained for the measured distance between the anchors and the tag compared to the calibration method that did not include orientation information. With the least-square estimator, 14 cm RMSE in 3D is obtained with the NN model corrected distances, with a 9 cm improvement compared to when raw distances are used. The method produces better results without the need to obtain the UWB module’s diagnostics parameters that are required to calculate the received signal strength or channel response, and in this way maintain the minimum packet size for the ranging protocol.

## 1. Introduction

Indoor localization using ultra-wideband (UWB) radio is a popular research topic [1,2,3,4,5,6] and an off-the-shelf real-time localization system [7]. Its suitability for mobile applications and its great localization accuracy provide solutions for a wide spectrum of applications. In real-time localization systems (RTLSs), the tags (agents, mobile units) are localized by measuring the distances from the tag to the anchors (ground station, static units) with known positions. The distances between the anchors and the tags can be measured with the time-of-arrival (TOA) [8] or the time-difference-of-arrival (TDOA) [9] methods. The accuracy of the measured distances depends on the accuracy of the tag’s position [10]. An orientation-dependent error is present in UWB-radio distance measurements [11,12,13,14,15]. The speed of propagation in various materials around the antenna’s electrical center point cannot be empirically determined. The error is a superposition of multiple factors: antenna design and its radiation diagram; the electronic circuit build around the antenna and the radio module; the protective housing and its dielectric properties. A non-empirical model that would contain a large set of distance and orientation variations could be relevant for effective error mitigation.

In [16], the authors presented a time-difference-of-arrival (TDOA) localization system for monitoring elderly people utilizing UWB technology, presenting 1 m error for stationary measurements. In [17], UWB technology combined with motion capture is used for the localization of people and goods at an industrial site with 0.15 m root mean square error (RMSE) in 2D space. Another case of UWB real-time localization systems (RTLSs) used in an industrial environment is presented in [18], where authors achieved 2D positioning errors from 0.02 m to 0.38 m in six tag positions. Tieman et al. [19] presented a UWB time-of-flight-based localization system for parking an electric vehicle on a dedicated recharging parking lot. With only two anchors an error of less than 10 cm was obtained in the experiments.

Much work was carried out to improve distance measurements between radio modules with the use of machine learning. In [20], the authors used a neural network (NN) with a received-signal-strength indicator (RSSI) and an RGB image as the input to produce the estimated distance between two modules with greater accuracy in non-complex environments without buildings. Authors achieved 0.5 m ranging errors in open scenarios and under 0.9 m ranging errors in more complex scenarios with objects in proximity of modules. Chen et al. [21] presented a ranging model based on a backpropagation NN (BNN). The model showed better performance in simulations than shadowing models and the capability to adapt to a specific environment with an average ranging error of 0.34 m. Schmid et al. [22] investigated machine learning approaches in error mitigation using additional diagnostics data and without the tag’s orientation. Their goal was to reduce the error due to multipath effects. With an artificial NN and 10 input parameters obtained from the modules, they were able to reduce the mean absolute ranging error from 0.08 m to 0.035 m. With histograms of errors, they showed that the error could be reduced to a smaller extent with only two out of 10 input parameters, the measured distance and the maximum observed noise during the first path detection.

In previous work, an orientation-dependent distance error was observed when measuring the distance between two UWB modules. In [11], the authors proposed a method to correct the positioning error due to the antenna’s orientation in a time-difference-of-arrival (TDOA) localization system. In simulations with line-of-sight (LOS) conditions and 1.24 mm ranging noise, they achieved a sub-mm (0.1 mm) range RMSE with 10 base stations. A configuration with a smaller number of base stations did not converge to the mm-range RMSE. The authors in [12] observed the effect of a custom UWB antenna’s orientation when ranging between two modules in a TOA system. In experiments, they measured the antenna in five different orientations for two planes and observed different distance errors for different antenna orientations at 0.5- and 1-m distances between the receiver and transmitter antennas. The minimum error obtained was 0.001 m, and the maximum error was 0.045 m. In [13], the authors presented experiments in a vehicle with transmitting antenna in four different orientations. The effects of the antenna orientation and the multipath environment were analyzed through the channel impulse response (CIR). A linear piecewise model of the power-delay profile in logarithmic scale and a generalized extreme value model of a small-scale digital fading are presented. Bregar et al. [14] used two methods where the CIR is used with two convolutional neural networks (CNNs) for non-line-of-sight (NLOS) classification and ranging-error-regression modeling. They showed that ranging-error regression performs better than filtering the NLOS ranges and that combining the methods improves the performance compared to the least-squares (LS) and weighted LS methods. With 10 anchors and a weighted LS location estimator with ranging error mitigation, they achieved a mean localization error of 0.113 m.

The authors in [15] presented an orientation-dependent neural network model for error mitigation. They experimentally determined and analyzed the error through the channel response from two experiments: indoor and outdoor. The authors observed the optimal mode of operation for the selected UWB module where the orientation-induced error is the smallest. The neural network is trained with channel-response data. Using the trained model on the data that were not used in the training process, ranging errors of less than 1 cm were obtained for the optimal mode of operation.

A novel, simplified method to improve the distance measurement with a UWB radio used in real-time localization systems is proposed in this paper. The presented model is heavily simplified compared to previously used NN models for mitigating orientation-dependent ranging error [15] in NN architecture and input parameters’ complexity. Only two parameters are used for the distance corrections: the measured distance between the anchor and the tag, and the tag’s orientation. These parameters are fed to an NN, previously taught with a learning dataset consisting of distance measurements between two UWB modules at different distances and with different orientations. The trained model in this work is first evaluated with data from the training-measurement campaign that were not used in the training process and then in the experimental setup of 12 anchors and one tag in six different poses, presenting the model results in more realistic conditions. The results show that the used model improved the overall distance measurement in 3D space. Consequently, the localization of the tag is improved.

In the first part of the paper, the RTLS system design, training-data-measurements equipment and procedure, the NN model, the RTLS setup with 12 anchors and the reference measurements for the evaluation are described. Then, all the results from the training measurement and the RTLS experiment are presented in the results section.

## 2. Materials and Methods

### 2.1. System Design

**Figure 1:**
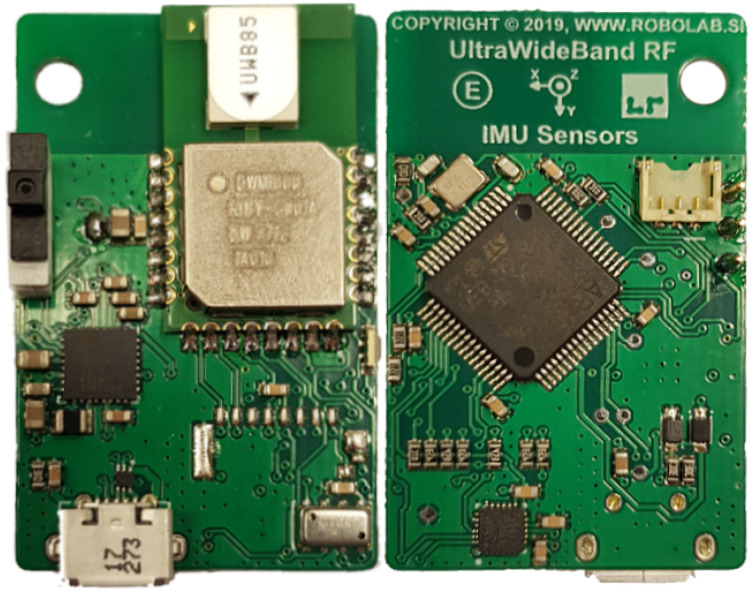
Top and bottom sides of the designed board with the ultra-wideband (UWB) radio module.

**Figure 2:**
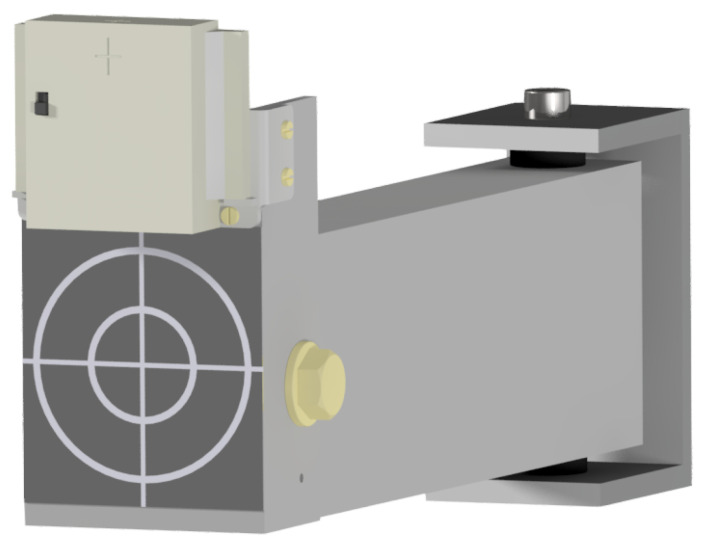
UWB module in a plastic housing as it is mounted on the wall with the mount. Under the module is a target for the geodetic reference measurements of the anchors’ positions. The relationship between the antenna center and a geodetic target is known.

The real-time localization system consists of 1 tag and 12 anchors to ensure dense enough placement of anchors in the RTLS system in an experimental environment. Each module consists of a printed-circuit board with ultra-wideband (UWB) DWM1000 modules for ranging, an STM32L4 microprocessor and a USB port for power supply and data transfer to the computer for further processing (Figure 1). The modules are enclosed in a protective ABS plastic housing designed to be fixed to the wall with custom mounts (Figure 2).

**Figure 3:**
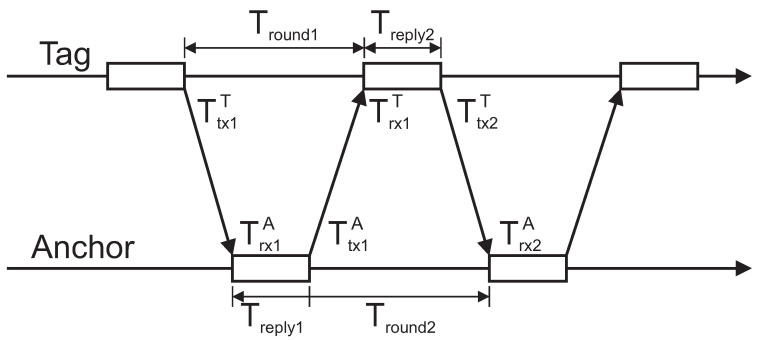
Double-sided two-way ranging with four messages. In this way, all the timestamps for the time-of-flight calculations are collected on the tag side.

For distance measurements, the double-sided two-way ranging version with four messages is implemented on the UWB modules [23]. Implemented ranging method reduces error due to the clock drift on both devices and does not require symmetrical reply delays. The scheme (Figure 3) represents one ranging cycle required for calculating the distance, where the tag initiates the ranging and all the timestamps are obtained on the tag’s side with the fourth packet.

The time of flight can be calculated with:

(1) $${\hat{T}}_{TOF}=\frac{T_{round1}\times T_{round2}-T_{replay1}\times T_{replay2}}{T_{round1}+T_{round2}+T_{replay1}+T_{replay2}},$$

where

(2) $$\begin{array}{cc} T_{round1} & =T_{rx1}^{T}-T_{tx1}^{T}\\ T_{round2} & =T_{rx2}^{A}-T_{tx1}^{A}\\ T_{reply1} & =T_{tx1}^{A}-T_{rx1}^{A}\\ T_{reply2} & =T_{tx2}^{T}-T_{rx1}^{T}. \end{array}$$

The distance between the tag and the anchor is then:

(3) $$d={\hat{T}}_{TOF}\times c,$$

where *c* is the speed of light. The distance is calculated on the module and sent to a connected device over a USB serial port.

For sending distance, 8 bytes of a payload are required. Diagnostics data for power calculation take additional 128 bytes and CIR data another 4064 bytes, of which half is for real and a half for the imaginary part. Altogether payload would be 4200 bytes long, of which diagnostics and CIR data represent 99.8% for the selected hardware. Additional bytes in the payload drop the data rates 10-fold.

### 2.2. Neural Network Model and Training Data Measurements

**Figure 4:**
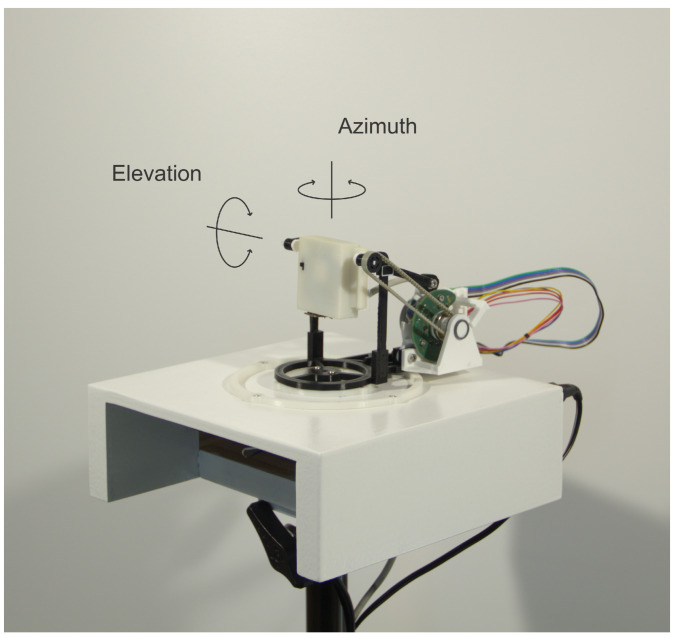
Gimbal mechanism for rotation of the UWB module around the azimuth and elevation axis centered in the antenna phase center with stepper motors and RLS Orbis absolute rotary encoders.

For collecting the training dataset needed for the NN’s training, the gimbal was designed (Figure 4). The gimbal enables the rotation of the UWB module around the azimuth and in the elevation plane relative to the antenna center with the USB cable connected to the module for the power supply and data transfer. For axis positions, two absolute rotary encoder RLS Orbis were used. To minimize the volume of any material around the UWB antenna, an elevation motor was placed behind the module, as low as possible. The motor is then connected to the gimbal axis via a timing belt. Position control is implemented on a microcontroller and can be driven with a PC over a USB cable.

**Figure 5:**
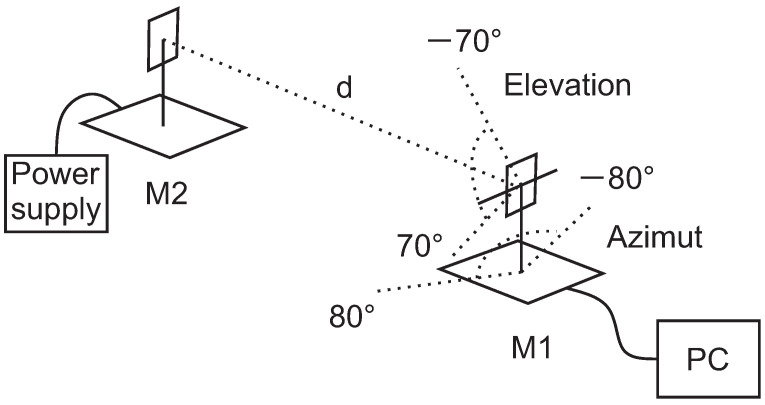
Setup for training-data measurements with module M2 on a stand and module M1 on a stand with the gimbal. For M1, the range of motion for the elevation was from −70 to 70 degrees and for azimuth, from −80 to 80 degrees.

The training dataset was measured with two UWB modules that were not used in the RTLS setup. One module was stationary on a stand and connected to a power supply. The second was attached to the gimbal to rotate the UWB module around the azimuth and elevation angles around the antenna’s center (Figure 5). The reference distance between modules was measured with a laser distance meter with an accuracy of 1 mm.

**Figure 6:**
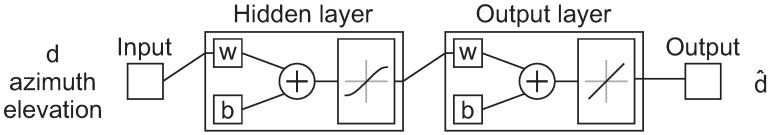
Feedforward neural network with six neurons in the hidden layer. The neural network (NN) has three input parameters—measured distance, azimuth and elevation angles, and one output parameter—corrected distance.

Measured and reference distances, azimuth and elevation angles were then used for the training of a feedforward neural network. The Matlab Deep Learning Toolbox was used to design and train the model. A feedforward neural network with six neurons in the hidden layer was chosen, with hyperbolic tangent sigmoid transfer function in the hidden layer and linear transfer function in the output layer (Figure 6). The process of choosing the number of neurons in the hidden layer is described in the result section.

### 2.3. Reference Measurements for NN Model Evaluation

The trained NN model for the distance correction was evaluated experimentally with the RTLS in the laboratory. In this case, for the reference distances between the anchors and the tag, the reference system Optotrak Certus and geodetic measurements were used. With Optrotak Certus, the tag’s reference position and orientation were measured with three-point markers attached to the tag. The uncertainty of the position measurements after multiple Optotrack cameras were registered was less than 1 mm.

The 12 anchors’ ground-truth positions were measured with a LeicaTPS 1201+ certified electronic tachymeter, representing the standard in reference-position measurements. The wall-mounting plates for the anchors were designed in such a way that the reflective targets for the geodetic measurements could be fixed to them, with a known offset between the target center and the UWB antenna. The offsets between the coordinate of the reflective target’s center and the anchor’s antenna were applied to the reflective target’s coordinates to obtain the true reference coordinates. The reference coordinates of the reflective targets were computed with an uncertainty of less than 1 mm.

For aligning both reference systems, shared reference points were measured with both systems. After the alignment, all the measured reference points with the Optotrak were in the same coordinate system as the anchors’ reference positions [24].

As stationary measurements were acquired in the RTLS experiment, the tag’s orientations were obtained with reference measurements. The orientation was calculated with three-point markers.

### 2.4. RTLS Setup for the NN Model Evaluation

**Figure 7:**
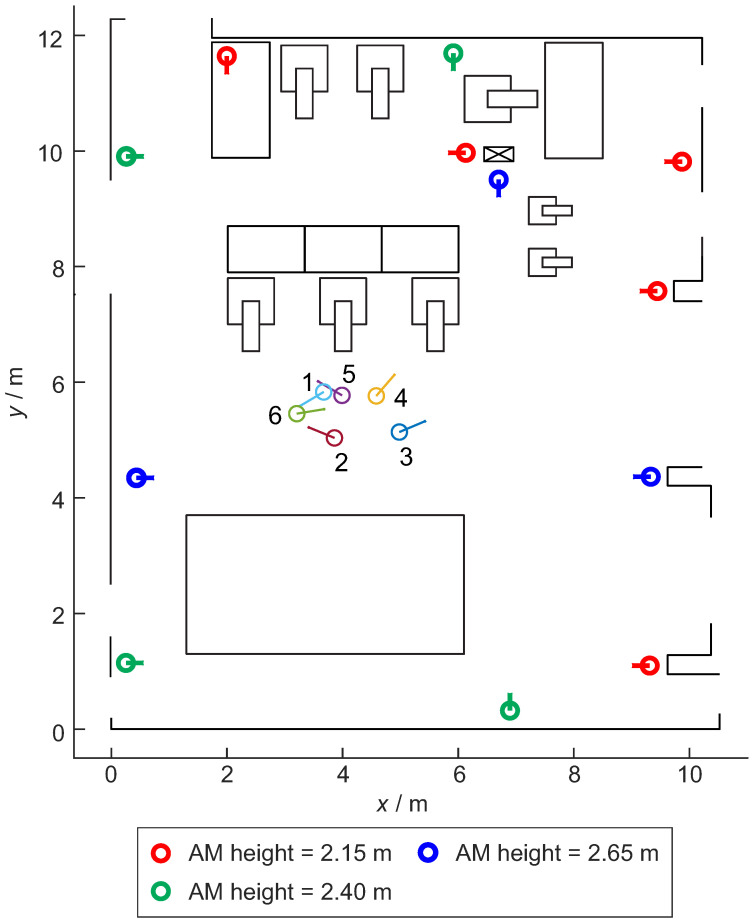
Real-time localization system (RTLS) setup in the laboratory with 12 anchors and 6 tag poses. The position for each pose is represented by a circle and the orientation with a short line. The short line represents the UWB zero-degree orientation. The same is valid for the anchors. The additional elements in the figure are other objects in the laboratory. AM heights in the legend describe the height of the anchor from the floor.

The RTLS setup with 12 anchors presented in Figure 7 was designed to evaluate the trained NN model in real conditions. The distances to all 12 anchors and the reference pose were measured with the Optotrak system for each tag’s pose.

**Figure 8:**
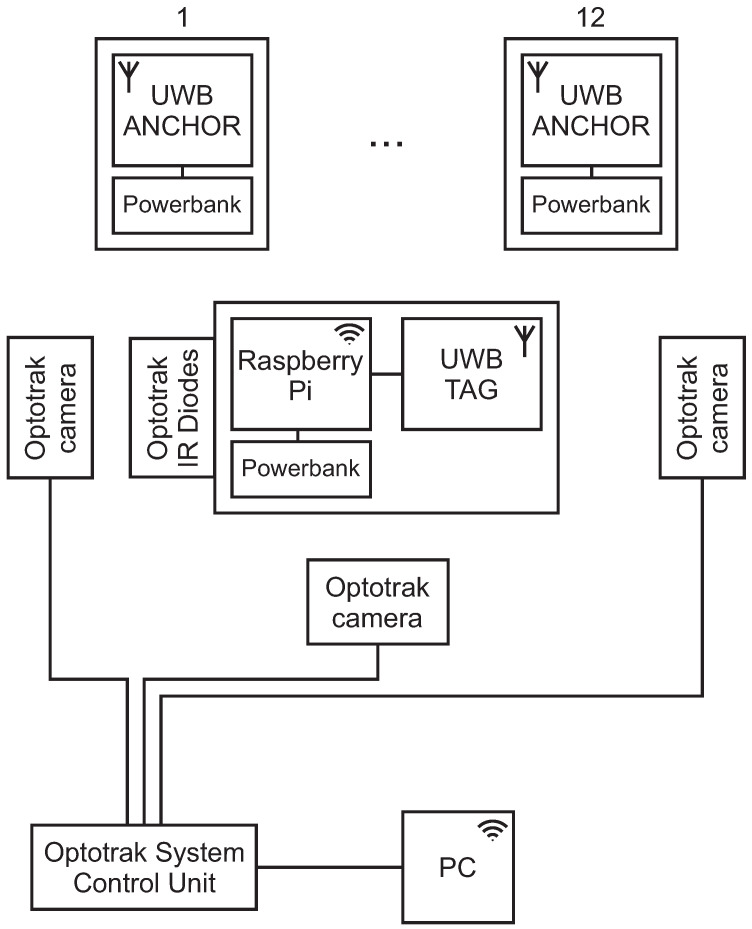
Experimental setup of 12 anchors and 1 tag for the model evaluation with the reference system Optotrak.

The anchors communicate with the tag only wirelessly, so just a power connection is provided to them with power banks (Figure 8). The tag is connected to a Raspberry Pi for the power and data transfer. The measured values are sent to the Raspberry Pi, where all the calculations are made. On the Raspberry Pi, a server is established so that the measured data can be transferred to a PC with Matlab Simulink. The Optotrak reference-position points are also acquired over a Simulink in the same scheme, so data synchronization is ensured at the data-acquisition level.

In the RTLS experiment, the UWB module with Optotrack IR diodes was placed at a selected position with a selected orientation. Then, the RTLS application was started, so the distances between the tag and the anchors were being measured and transmitted to the Raspberry Pi. Next, the Simulink scheme on the PC was started to acquire the measured distances between the anchors and the tag from the Raspberry Pi server. Simultaneously, the reference positions of three Optotrak IR diodes were gathered in the same scheme. After 250 samples were measured, the Simulink scheme was stopped and the procedure was repeated for the next tag’s pose.

**Figure 9:**
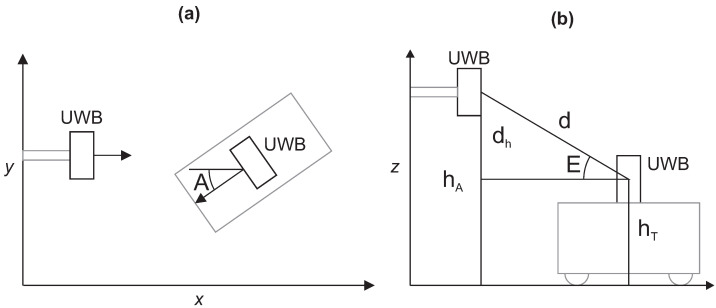
Tag’s azimuth and elevation relative to the individual anchor–tag pair. (**a**) The azimuth is computed from the anchor’s known orientation and the measured tag’s orientation around the *z*-axis in the reference coordinate system. (**b**) The elevation is obtained from the known heights of the anchor and tag and the distance between them.

For the orientation of the tag, the reference measurements from the Optotrak system were used. From three markers attached to the tag, the orientation in the reference coordinate system was calculated. The anchors’ orientations were known from the setup as marked in Figure 7 and are constant relative to the reference coordinate system. The azimuth estimation for the model input was computed from the tag and anchors’ orientations around the *z*-axis (Figure 9a). An elevation estimation was computed from the known anchors’ heights and the tag’s positions:

(4) $$\begin{array}{c} d_{h}=h_{A}-h_{T}\\ E=asin(\frac{d_{h}}{d}), \end{array}$$

where $h_{A}$ is the height of the anchor, $h_{T}$ the height of the tag and *d* the distance between the anchor and the tag (Figure 9b).

### 2.5. Method for Range Bias Distance Correction without the Tag’s Orientation Information

In cases where better accuracy is required in RTLS applications, additional corrections of the measured distance are recommended by UWB-radio manufacturers. In the presented work, a method that does not include the tag’s orientation information is used. It is called a range bias, which is the error induced due to the received signal power level. Tables are provided where the distance correction is mapped with the received signal power level and are translated to the measured distances [25]. The measured distance is directly used with the correction tables, from which the predicted error is selected for the measured distance.

## 3. Results

The following sections present the experimental results. First, the acquired data used in the NN model training are shown. Second, the results from choosing the number of hidden neurons in the NN model. Third, the results from the NN model with the reduced training data are presented to evaluate the impact of the amount of data on the results. Finally, the NN model results for the elevation/azimuth data that were not used in the NN model training and the data from the RTLS experimental setup are presented.

### 3.1. Training Dataset

**Figure 10:**
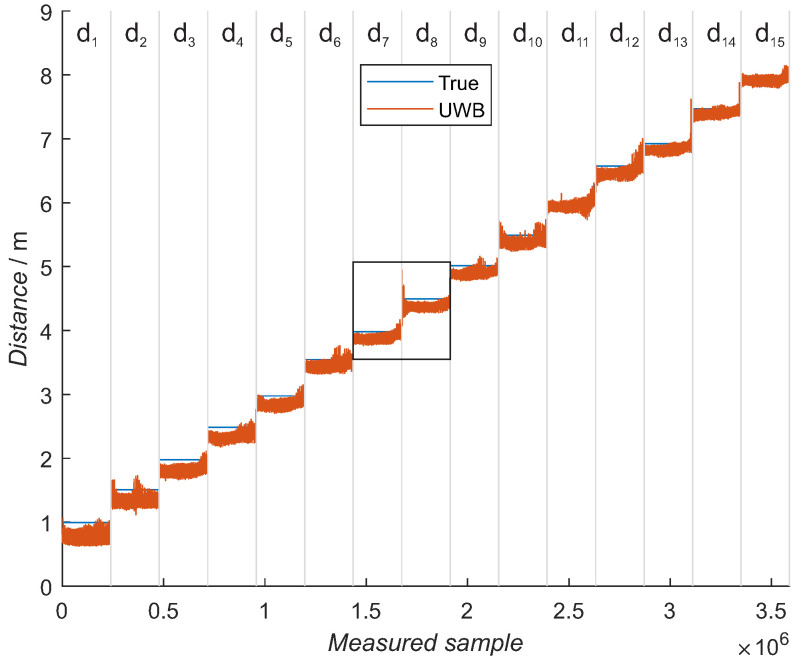
Training dataset with 15 different distances. For each distance, 33 azimuth and 29 elevation points were measured, and for each point, 250 samples were measured. The blue line represents the reference distance. Each distance between the UWB modules is separated with gray vertical lines. The black rectangle shows zoomed distances presented in separate figures.

**Figure 11:**
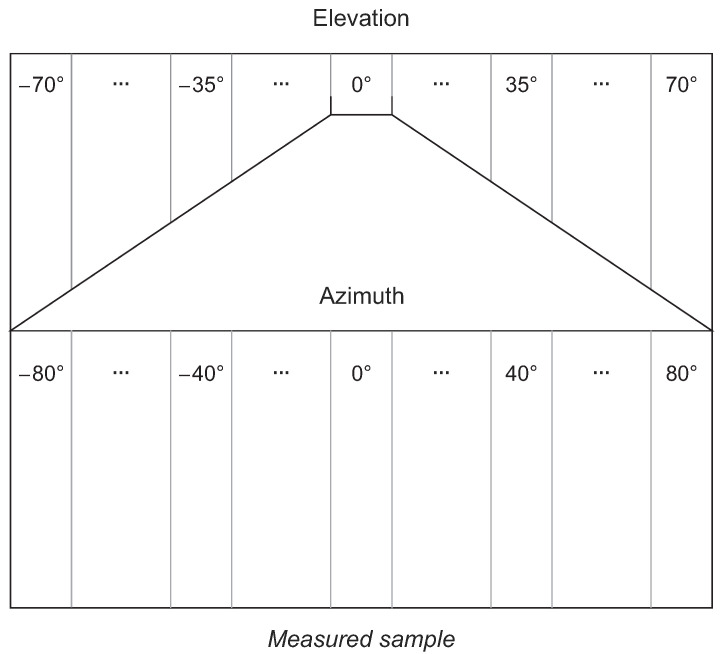
Presentation of the measured training data inside one distance between the UWB modules. The elevation was changing from −70 degrees to 70 degrees with a step size of 5 degrees, with the central position at 0 degrees. The azimuth was changing from −80 degrees to 80 degrees with a step size of 5 degrees, with the central position at 0 degrees inside each constant elevation.

The training dataset was measured at 15 different distances from 1 m to 8 m in 0.5-m increments, marked with the gray vertical lines (Figure 10), where the blue line represents the reference distance and the orange, the measured distance. For each distance, 29 elevations and 33 azimuth positions were measured, resulting in 957 angular values. For each angular value, 250 repetition samples were measured. Training dataset parameters are presented in Table 1. Each distance contains distance measurements where the elevation changes from −70 degrees to 70 degrees from left to right in 5-degree steps. The azimuth was changing from −80 degrees to 80 degrees in 5-degree steps from left to right inside each constant-elevation value (Figure 11), representing one azimuth sweep. When the elevation and azimuth values were 0 degrees, the UWB modules were oriented face to face. A similar trend is seen for each distance, where the distance is varying with elevation, with the lowest measured distances at central elevations. There are some exceptions with increased values at the edges of the elevation. Multiple sets of training data were measured. All the sets had similar measured distances for all the azimuth and elevation values and for all the selected distances.

**Figure 12:**
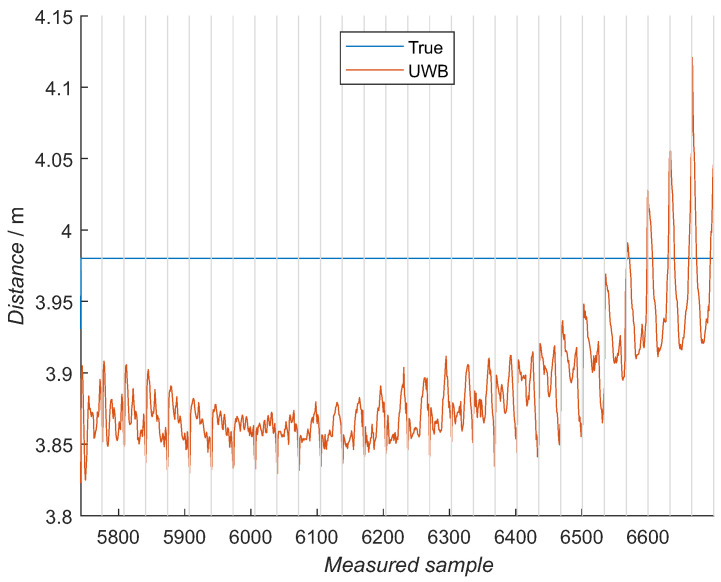
Zoomed training data acquired at 4-m distance, where a mean of 250 samples is presented for each azimuth and elevation point. Each gray compartment represents one azimuth sweep at a constant elevation, meaning the elevation is changing from −70 degrees on the left to the 70 degrees on the graph’s right-hand side. In each compartment, the azimuth goes from −80 degrees on the left-hand side to the 80 degrees on the right-hand side. The blue line represents the reference distance.

**Figure 13:**
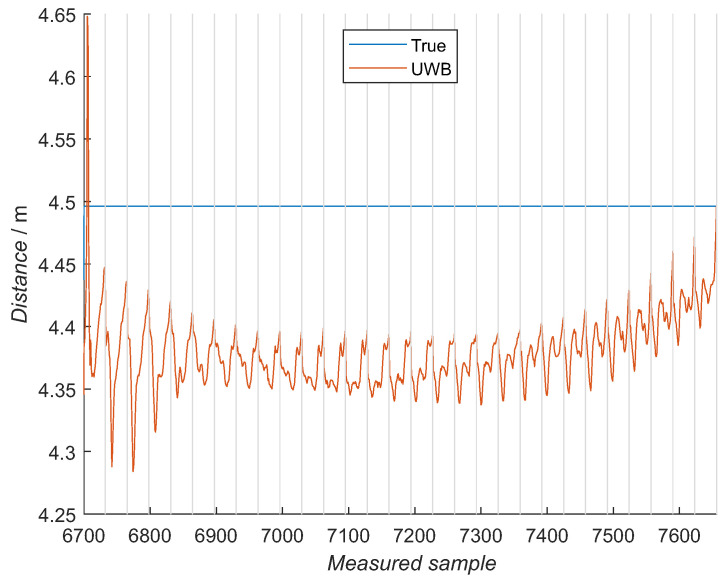
Exemplary set of zoomed training data for measurements at 4.5 m, where a mean of 250 samples is presented for each azimuth and elevation point. Each gray compartment represents one azimuth sweep at a constant elevation, meaning the elevation is changing from −70 degrees on the left to the 70 degrees on the graph’s right-hand side. In each compartment, the azimuth goes from −80 degrees on the left-hand side to 80 degrees on the right-hand side. The blue line represents the reference distance.

The mean of 250 samples for each angular value showed a clearer image of how the measured distance is changing with the elevation and the azimuth, Figure 12 and Figure 13 for 4 and 4.5 m, respectively. A pattern is present in the graphs, continuously changing from the left-hand side (−70 degree elevation) to the right-hand side (70 degree elevation). An average of 5-cm distance changes are present in one azimuth sweep at central elevation values with increased values of 25 cm within one azimuth sweep in elevation at the edges.

### 3.2. Data from RTLS Experiment with 12 Anchors

In the RTLS experiment, 12 anchors were used with the stationary tag in 6 different poses (Figure 7). Six different tag poses were selected to test multiple conditions, representing possible situations for mobile robots servicing stations up and down from the central area. For each tag’s pose, 12 distances were measured to each anchor–tag pair, and 250 samples were sampled for each distance. Six different tags’ poses resulted in 72 azimuth–elevation combinations with an angle range of 328 and 62 degrees for the azimuth and elevation, respectively (Table 2). Distances between the tag and anchors varied from 3 m to 8 m. In the next sections, results using measurements from the RTLS experiments are presented.

### 3.3. Number of Hidden Neurons

**Figure 14:**
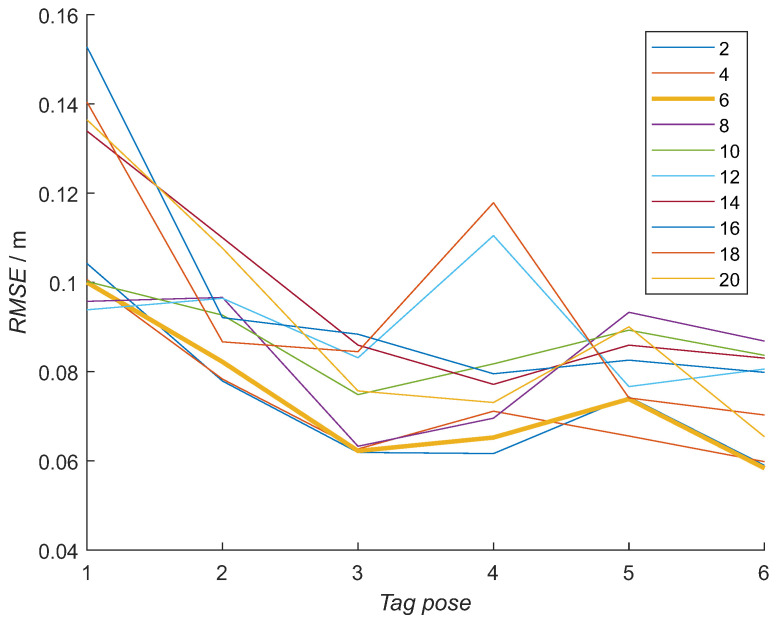
Results from neural networks with a different number of neurons in the hidden layer, all trained with the same training data. The RMSE is displayed for each tag’s pose. The numbers in the legend represent the number of used neurons in the hidden layers.

**Figure 15:**
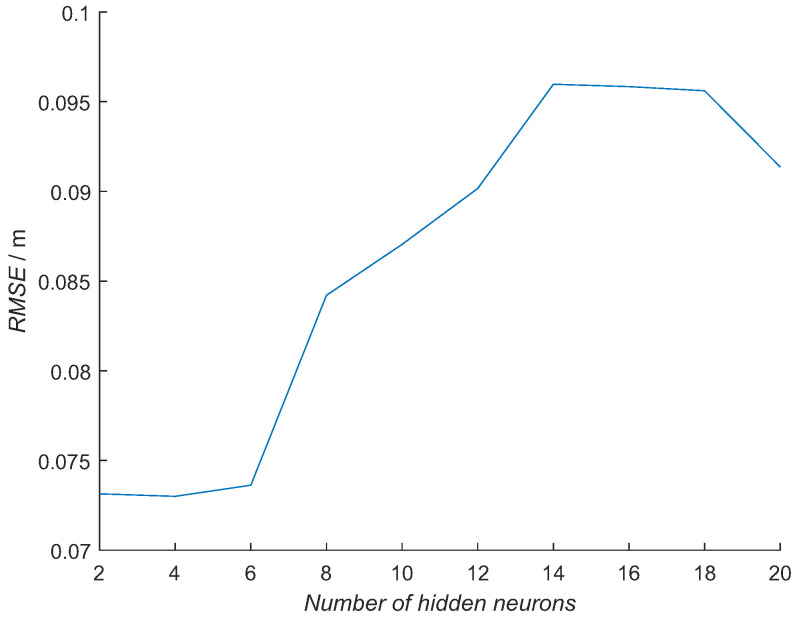
Mean error over all six tags’ poses for the NN models with the number of neurons from 2 to 20, with increments of 2 using the data from the RTLS experiment.

The optimal number of hidden neurons was determined experimentally. Ten different NN model configurations with the number of hidden neurons from 2 to 20 in increments of 2 were trained 10 times. For each NN model out of 100 and each tag pose, the RMSE was calculated using measured distances from the RTLS experiment. The NN model with the minimal average RMSE was selected for each number of hidden neurons from 10 trained NN models, obtaining the best NN model for each configuration (Figure 14). Then, from previously selected best-NN models for each configuration, the average RMSE for all the tag poses was computed for selecting the best NN model configuration (Figure 15 and Table 3 top row). In Figure 14, the RMSE is presented for each tag pose. In Figure 15, each number of hidden neurons is presenting an average of all six tag-pose RMSE values.

The RMSE with RTLS data increases with the number of hidden neurons, with the big step of 1 cm from 6 to 8 hidden neurons. The smallest errors are for the NN models with 2, 4 and 6 hidden neurons, where there is almost no difference among them. The average training times for each NN model configuration are presented in Table 4. The training times increase with more hidden neurons.

**Figure 16:**
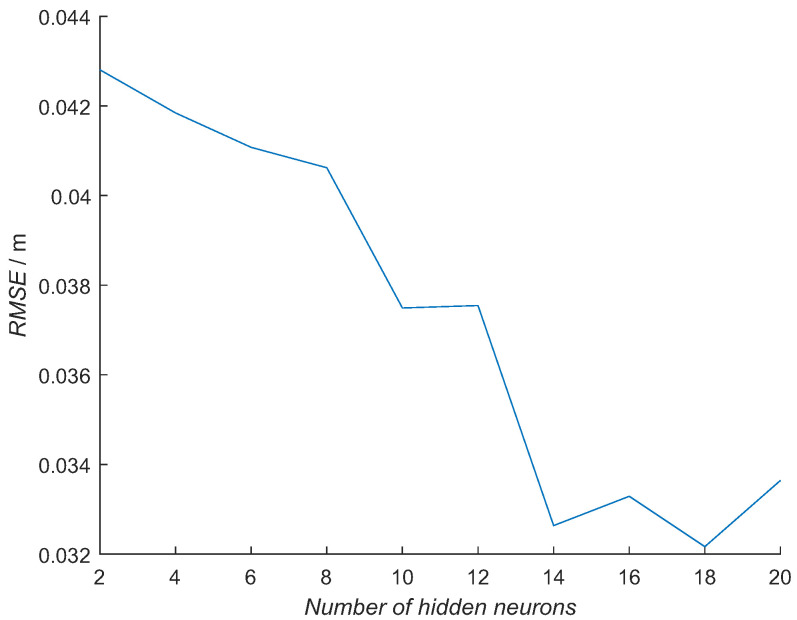
Mean error over all six tags’ poses for the NN models with the number of neurons from 2 to 20 with increments of two using data from the training experiment, that were not used in the NN’s training process.

The same process was repeated with a subset of data from the training experiment that was not used in the learning process (Figure 16 and Table 3 bottom row). The error with the subset of training data is decreasing with the increasing number of hidden neurons. The smallest errors are for NN models with 14, 16, 18 and 20 hidden neurons. Please note that the smallest RMSE values here are in the range of 0.033 m, while the smallest values in the RTLS experiment are 0.073 m.

### 3.4. Reduced Learning Dataset

**Figure 17:**
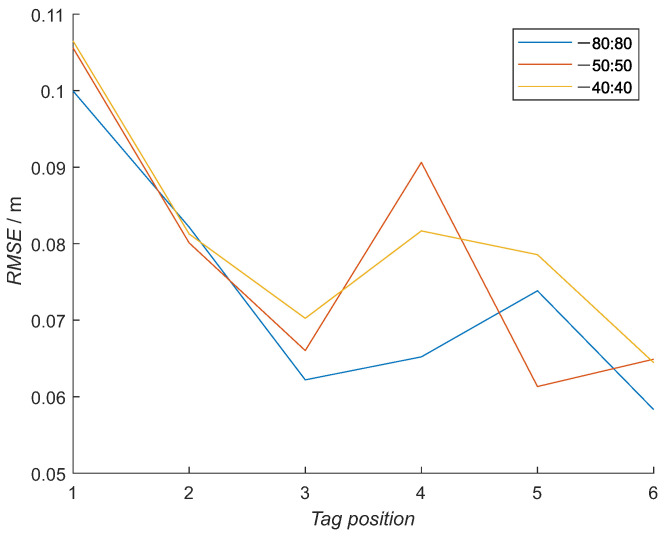
RMSE for all six tags’ poses with reduced training data for the neural network. From original −80 to 80-degree elevation span, two smaller data sets were made: from −50 to 50 and from −40 to 40-degree elevation span.

For example, in Figure 12 and Figure 13 can be seen increased distance values for low and high elevation angles, which is the most left and right in the figures. These values can represent the actual situation of the UWB module or are caused by outside parameters present during the training experiment. To test this, the elevation range was first reduced to −50 degrees to 50 degrees span and further to −40 degrees to 40 degrees span. Overall, the NN model with a full range learning dataset obtained the smallest RMSE (Figure 17), where the mean values over all the tag’s poses are 0.074, 0.078 and 0.081 m for the full dataset, −50 degree to 50 degree span data set and −40 degree to 40 degree span dataset, respectively (Table 5). The NN model with the full training data is, in all tag poses, better or equal to the reduced model, except in the fifth tag pose, where the least-reduced model has a smaller error.

### 3.5. Model Results

Finally, the output of the trained NN model with six hidden neurons (Model) was compared with a compensation method (Range Bias) that is not incorporating the tag’s orientational information and the raw distance measurements (Raw).

First, methods are used on the subset data from training measurements that were not used in the NN model’s training process. Mean errors were 0.041 m, 0.315 m and 0.132 m for the NN model, Range Bias and Raw measurements, respectively. With the subset data, the error is improved by 9 cm with the presented model. The non-orientational range-bias method increased the error by 18 cm.

**Figure 18:**
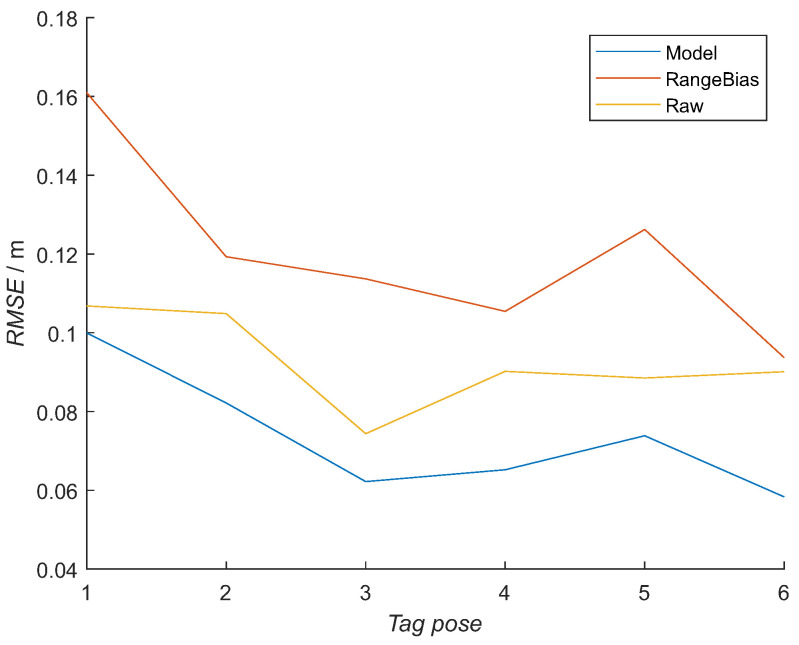
RMSE error for distances to all 12 anchors in the RTLS realistic setup in a laboratory for all six tags’ poses for the presented model (Model), a method that does not include orientational information (Range Bias) and raw measurements (Raw).

All three methods are used on data from the RTLS measurements in the laboratory (Figure 18), presenting the realistic conditions with obstacles and multipath situations. The NN model improved the mean error over all six tags’ poses by 2 cm. The RMSE was improved for all six poses, finally having a maximum error of 0.1 m (Table 6). The non-orientational method did not improve the accuracy of the measured distances, having a 3-cm larger mean error than the raw measurements.

## 4. Discussion

Previous work presented an antenna-orientation-induced error in ranging with UWB radios as well as a model for ranging-error prediction. Generally, all the models used a channel impulse response or received the signal power as inputs, enabling a good error-prediction model. This approach makes the model more complex and requires additional data from the modules. This paper presents a simple ranging-error model with only distance, azimuth and elevation as the input, which improves the measured distance in real-time localization systems in a realistic situation, in 3D space.

For training NN models, the training data were first acquired at different distances and orientations between two UWB modules. Patterns can be observed in the measured values, for the elevation and azimuth observations (Figure 12 and Figure 13). One azimuth sweep is made for one constant elevation value. Peak values of the measured distances are present at the edges of and in the middle of the azimuth sweep. Although the shapes are not the same for all the elevation values, the neighboring azimuth sweeps have a similar shape. This pattern is changing when changing the angle of elevation. The most significant changes are present in the boundary-elevation values with the greatest distance deviations within one azimuth sweep. When observing the elevation changes, the hyperbolic shape is seen in the measured values with the largest measured distances at the boundary angles when the UWB module faced the floor and at the ceiling. Multiple, very similar training datasets were measured with little difference between them, showing repeatability of the distance measurement with a designed gimbal and measuring procedure. This result is confirmed by evaluating the NN model with a data subset from training, where an RMSE error of 4 cm was achieved. Larger distance deviations at the elevation-boundary values are further discussed later on with additional NN models.

Based on the results presented in Figure 15 and Figure 16, the NN model with six hidden neurons was selected for further evaluations. The error is increasing with a larger number of hidden neurons in the NN model when using the RTLS experimental data (Figure 15). RMSE has a similar value for the NN models with 2–6 hidden neurons with a sudden increase for 1 cm with an increase of the hidden neurons to 8. This suggests that more neurons do not add to the model’s capability to describe an orientation-dependent ranging error in real conditions. The opposite is happening with an error when the training data subset is used (Figure 16). The error is slowly decreasing with an increasing number of hidden neurons. This is an expected result, as a more complex NN model can model the error better when similar data are used for the evaluation. In this case, more complex NN models perform better. Although the NN model with a larger number of hidden neurons had the smallest error with the training-data subset, they did not perform better in real-scenario measurements. The best NN-model candidates for the real environment were the NN models with 2, 4 and 6 hidden neurons, as they had a similar error in the RTLS experiments. This was achieved for shorter training times (Table 4). The NN model with six neurons was selected due to a slightly smaller error with the training data subset.

Training data have occurrences of longer measured distances at the boundary-elevation values for all the repeated sets. This means that the propagation times were the longest at these angles. Additional NN models with reduced training measurements were made to test whether longer distances at the edges of the elevation values are caused by the UWB module or by the gimbal and the testing environment. The training data were reduced to the elevation ranges −50 degrees to degrees 50 and −40 degrees to 40 degrees. When new NN models were used on the RTLS data, the same or larger errors were computed when observing the mean error over all six tags’ poses (Figure 17). If the errors would be smaller than the error from the full range NN model, the training data would be influenced by factors not solely caused by the UWB module. The results confirm the validity of the training data and the process of measuring the training data.

The performance of the new NN model, Range Bias and the Raw data is 0.041 m, 0.315 m and 0.132 m, respectively, where the distance measurements’ subsets from the full training experiments are used, which were not utilized in the NN training process. The error of 4 cm was achieved with a 69% improvement compared to the situation without the NN model. Compared to the results from previous research (Table 7) similar results were achieved. Schmid et al. [22] achieved slightly better results but with constant orientation between a pair of modules and additional diagnostics data. Tiemann et al. [15] had better results than the presented model and Schmid, where an approximately 1-cm error was achieved with the channel impulse response as the input. The presented model achieved a larger error compared to [15,22], but the model’s implementation is much simpler, with only distance as an input, in addition to the orientation, which is a common input for the presented model and [15].

Finally, the NN model of the orientation-dependent ranging error was evaluated in the RTLS experiment in realistic indoor conditions in 3D space. Overall, the NN model improved the measured distance in the RTLS system by 2 cm (Table 6). The non-orientation method that was used for comparison did not improve the measured distance. This is expected as the non-orientation method does not take a tag’s orientation into account. Correcting the distance only based on the measured distance can result in the wrong corrections and larger errors. The NN model was proved to improve the measured distance, even if not all possible orientations from the RLTS experiment were captured in the training data. This means that there is a possibility to improve further the overall measured distances with the proposed orientation-dependent ranging-error model.

Following the presentation of results in this paper, it could be claimed that the NN model is a simple ranging model suitable for RTLS applications with easy implementations and without additional parameters.

## 5. Conclusions

This paper presents a simple neural-network orientation-dependent ranging-error model in 3D space. The selected feedforward NN model required the measured distance and the calculated orientation, elevation and azimuth, as an input. The experimental data and the results when choosing the NN model configuration are presented and discussed. When testing a different number of neurons in the hidden layer on training data, NN model error decreases with a higher number of neurons. The opposite is with testing the NN model on RTLS experimental data, where error increases with an increasing number of neurons. Six neurons in the hidden layer were selected for the final NN model configuration. The training data are also validated with additional NN-model variations, where the boundary-elevation angles were checked with a reduced training dataset. NN model trained with the full range training dataset obtained the best results. The NN model’s performance is validated with a data subset from the training measurements that was not used in the training process, achieving a 4 cm error. Compared to the literature, the errors are not improved to the same extent, but the model’s complexity and additional requirements for acquiring the necessary input parameters are smaller in this case. Finally, the first demonstration of the orientational ranging-error model in the RTLS experiment with real conditions is presented, where the NN model showed improvements in 3D measured distances by 2 cm with a mean error of 7.4 cm. It should be underlined that the distances are estimated in 3D. In future work, extended training datasets will be tested and, consequently, the complexity of the training data vs. the distance improvement compared. New measurements are planned for testing the model in dynamic conditions. Further research should focus on investigating the use of additional NN model input parameters, e.g., speed of the user.

## Figures and Tables

**Table 1:** Training dataset parameters.

	Min	Max	Step
distance range/m	1	8	0.5
azimuth range/${}^{\circ }$	−80	80	5
elevation range/${}^{\circ }$	−70	70	5

**Table 2:** Min and max azimuth and elevation values for six tags’ poses in the RTLS experiment.

	Min	Max
azimuth/${}^{\circ }$	−157	171
elevation/${}^{\circ }$	−90	−28

**Table 3:** Mean error over all six tags’ poses for a different number of hidden neurons in the NN model. The first row, RTLS, presents model errors when measured distances from the RTLS experiment were used. The second row, SUBSET-TRAIN, presents model errors when a subset of data from the training campaign was used that was not included in the NN model’s training process.

Hidden Neurons	2	4	6	8	10	12	14	16	18	20
RTLS error/m	0.073	0.073	0.074	0.084	0.087	0.090	0.096	0.096	0.096	0.091
SUBSET-TRAIN error/m	0.043	0.042	0.041	0.041	0.037	0.038	0.033	0.033	0.032	0.034

**Table 4:** Average training time for NN models with a different number of hidden neurons.

Hidden Neurons	2	4	6	8	10	12	14	16	18	20
train time/s	280	487	795	1643	2173	2546	2489	3445	3774	3919

**Table 5:** NN models’ mean error with an elevation range of −80:80, −50:50 and −40:40 when RTLS experiment measurements were used.

Elevation Range/${}^{\circ }$	−80:80	−50:50	−40:40
mean error/m	0.074	0.078	0.081

**Table 6:** Mean, max and min error for all six tags’ poses for the presented model, non-orientational method and raw measured distances.

	NN Model	Range Bias	Raw
mean error/m	0.074	0.120	0.092
max error/m	0.100	0.161	0.107
min error/m	0.058	0.094	0.074

**Table 7:** Results from previous research with the use of NN in ranging. Error column represents ranging errors obtained with similar data as was used in NN training. The RTLS error column is presenting ranging errors obtained with NN models with RTLS measurements in realistic conditions.

	Notes	Error/m	RTLS Error/m
Wu et al. [20]	CNN-RSSI + Image *	0.5–0.9	/
Chen et al. [21]	BNN-ranging-simulations *	0.34	/
Schmid et al. [22]	NN-ranging + diagnostics data *	0.035	/
Tiemann et al. [15]	NN-ranging + diagnostics data	0.01	/
Presented model	NN-ranging; simple model, smaller payload	0.041	0.074

* No orientational data were used.

## Data Availability

All data are integral part of the correspondent author’s PhD thesis.

## Abbreviations

The following abbreviations are used in this manuscript:

BNN	Backpropagation neural network
CIR	Channel impulse response
CNN	Convolutional neural network
GPS	Global positioning system
LOS	Line-of-sight
LS	Least squares
NLOS	Non-line-of-sight
NN	Neural network
RMSE	Root mean square error
RSSI	Received signal strength indicator
RTLS	Real-time localization system
TDOA	Time-difference-of-arrival
TOA	Time-of-arrival
TOF	Time-of-flight
UWB	Ultra-wideband
